# The Effect of a Chemical Foaming Agent and the Isocyanate Index on the Properties of Open-Cell Polyurethane Foams

**DOI:** 10.3390/ma15176087

**Published:** 2022-09-02

**Authors:** Klaudia Kamińska, Mateusz Barczewski, Maria Kurańska, Elżbieta Malewska, Krzysztof Polaczek, Aleksander Prociak

**Affiliations:** 1 Department of Chemistry and Technology of Polymers, Faculty of Chemical Engineering and Technology, Cracow University of Technology, Warszawska 24, 31-155 Cracow, Poland; 2 Institute of Materials Technology, Faculty of Mechanical Engineering, Poznan University of Technology, Piotrowo 3, 61-138 Poznan, Poland

**Keywords:** polyurethane, open-cell foams, closed-loop economy

## Abstract

This article presents an ecological approach based on climate neutrality to the synthesis of open-cell polyurethane foams with modified used cooking rapeseed oils. Water was used as a chemical blowing agent in the amount of 20–28 wt.% in relation to the weight of the bio-polyol. The influence of water on the physical and mechanical properties of the synthesized foams was investigated. The resultant porous materials were tested for the content of closed cells, cell structure, apparent density, thermal conductivity, compressive strength, and dimensional stability. It was found that the apparent density decreased in the range of 11–13 kg/m^3^ when the amount of the foaming agent was increased. In the next step, a foam with a water content of 22% was selected as having the most favorable physico–mechanical properties among all the foams with various water contents. The isocyanate index of the selected foam was then changed from 0.6 to 1.1 and it was observed that the compressive strength increased by an average of 10 kPa. The thermal conductivity coefficients of the final materials with different water contents and isocyanate indices were comparable and in the range of 40–43 mW/m·K.

## 1. Introduction

Polyurethane (PU) foams are one of the most popular polymers in the world (in 2020, the global market of all PUs amounted to almost 24 million tons) and are usually obtained from petrochemical raw materials [1]. Polyols and isocyanates necessary for the synthesis of PUs, obtained through refining crude oil, contribute to the increase in greenhouse gas (GHG) emissions [2]. To remedy this, the current technological development aimed at protecting the environment is moving towards the use of renewable materials. Natural resources, applied both in thermal insulation and in usable materials, are becoming more and more important following the high cost of crude oil and the idea of sustainable development [2,3]. One of the strategies is the Circular Economy. First of all, its aim is to reduce the amount of waste produced, while the waste already generated should be used as secondary raw materials in the next production process [4,5,6]. However, given the technical limitations in obtaining the necessary energy to close the loop, it is necessary to consider a different material-efficient approach [7]. Therefore, the main goal of the European Commission, in line with the climate neutrality initiative, is to achieve a zero GHG economy by 2050 [8,9,10,11]. To keep the increase in global average temperature below 2 °C, more than 100 countries have approved reductions in greenhouse gas emissions. The industrial sector itself is responsible for the direct 21% of GHG emissions, however, as much as 31% of CO_2_ emissions are indirect emissions [12]. The reduction of GHG emissions can be achieved, e.g., by using renewable raw materials (including waste) in production and by improving the thermal conductivity of materials used in construction [3].

One of the main components of PU synthesis—polyols—can be obtained from raw materials of natural origin, including suitably modified post-frying vegetable oils. Examples of such oils are rapeseed, coconut, soybean, or sunflower oils that are widely used in European and Asian countries [13,14]. However, in order for vegetable oils to be used as bio-components for the production of polyol substrates, it is necessary to apply an appropriate chemical modification. Most often, the structures of vegetable oils do not contain hydroxyl groups, but double bonds are present, which allow for obtaining polyols as a result of appropriate chemical reactions [15]. The basic methods of synthesizing bio-polyols from fatty acids derived from vegetable oils are transesterification, ozonolysis, or epoxidation combined with the opening of oxirane rings [16,17].

The advantage of introducing the principles of climate neutrality into the synthesis of bio-polyols through the use of waste oils is the elimination of the problem of using cooking oil for the production of chemicals [4]. Such application of used oils, after the frying process, minimizes interference in food production, while reducing the emission of carbon dioxide to the atmosphere [5]. According to the data, described by A. Fridrihsone et al., the reduction of GHG emissions is approximately 1.40–2.79 kg of CO_2_ for 1 kg of bio-polyol produced on the basis of transesterified rapeseed oil for 1 kg of petrochemical polyol [18].

Rigid polyurethane foams (RPUF) are characterized by low apparent density, favorable thermal insulation properties, relatively high mechanical strength, and, most often, a closed-cell structure [17]. For these reasons, RPUF are used in many areas of industry, especially in construction. In addition to closed-cell PU foams, there are also foams with over 95% open cells. Due to vapor permeability, lower apparent density (7–14 kg/m^3^), and lower price compared to closed-cell foams, open-cell PU foams are used, e.g., in attic insulation [13,19]. In order to obtain an open-cell structure, the cell walls must be ruptured in the final foaming step [16]. For ecological reasons, blowing agents used for foaming should necessarily have a low ozone depletion potential (ODP) and global warming potential (GWP) [20]. Water can be used as an environmentally friendly blowing agent, however, its effect on open-cell PU foams has not been described in the literature so far [20,21,22]. It is important to select the appropriate amount, because the excess of a chemical blowing agent will contribute to larger and heterogeneous shapes of cells, resulting in increased thermal conductivity of PU materials [22].

The isocyanate (NCO) index is defined as the ratio of used isocyanate to theoretical amounts of isocyanate required. A change in the amount of water introduced to foam systems also influences the NCO index value [21]. A change in the NCO index value affects the PU foaming process itself and the mechanical strength of foams. To reduce the flexibility of PU foams, the NCO index can be increased, i.e., the content of rigid segments is increased due to the formation of additional urea bonds [23,24]. Typically, in industry, formulations of RPUF are in the range of the NCO index of 1.05–1.25, however, this applies to closed-cell foams [14]. So far, the influence of the NCO index on the properties of open-cell PU foams based on bio-polyol from rapeseed oil has not been described.

This article describes rigid polyurethane bio-foams that were obtained with the use of bio-polyols from used frying rapeseed oil. In order to develop appropriate foam recipes, the NCO index was modified. The paper assesses the effect of the amount of water and the NCO index on cellular structures and selected physical–mechanical properties, including thermal insulation properties, and dimensional stability of the open-cell PU foams. In the literature, there have been few studies on open-cell PUR foams, while the effect of the NCO index and foaming processes have not been described so far.

## 2. Materials and Methods

### 2.1. Materials

PU bio-foams were synthesized with the use of bio-polyol based on used cooking oil and triethylamine with a hydroxyl number of 360 mgKOH/g. The bio-polyol was obtained in accordance with the previously described transesterification method elaborated at the Cracow University of Technology [19]. Polymeric 4,4’-diphenylmethane diisocyanate (PMDI) with a free isocyanate groups content of 31 wt.% was supplied by Minova Ekochem S.A. (Siemianowice Śląskie, Poland). An amine catalyst (Polycat 142), emulsifier (Dabco EM400), and three surfactants (Tegostab 8870, Tegostab 8523, and Ortegol 500) were supplied by Evonik Industries AG (Essen, Germany). LANXESS (Cologne, Germany) supplied a flame-retardant—triethyl phosphate. Municipal water was used as a chemical blowing agent, which, in reaction with isocyanate, causes the formation of carbon dioxide. The recipes used for the preparation of open-cell PUR foams are summarized in Table 1 and Table 2.

### 2.2. Preparation and Samples

In order to obtain open-cell PUR foams, a one-step method was used with the use of two-component systems (component A—polyol masterbatch and component B—isocyanate). In order to homogenize component A containing the bio-polyol, catalyst, surfactants, emulsifier, chemical blowing agent, and flame-retardant, all the constituents were mixed for about 20 s. Then a specific mass of isocyanate (component B) was added to component A and the composition was mixed for 7 s. The systems were poured into open molds to allow the foam to rise freely in a vertical direction. The materials were left for 24 h at room temperature, and then cut into samples of appropriate dimensions required for tests. In the present work, the WX_Y sample labels were assigned according to the following rules: X—the mass of water in relation to 100 g of the bio-polyol used, and Y- the value of the isocyanate index.

### 2.3. Methods and Testing

The foaming process was analyzed using the foam qualification system FOAMAT (Format Messtechnik GmbH, Karlsruhe, Germany), which allows determining characteristic parameters, such as the reaction mixture temperature, pressure, and dielectric polarization, during the foaming process.

The Fourier transform infrared spectroscopy (FT-IR) measurements were realized using a spectrometer Jasco FT/IR-4600 (Japan, Tokyo) at room temperature (23 °C) in a mode of Attenuated Total Reflectance (ATR-FT-IR). A total of 64 scans at a resolution of 4 cm^−1^ were used in all cases to record the spectra. Additional analysis realized in order to calculate the degree of phase separation was based on measurements taken in the range between 1780 and 1640 cm^−1^ prepared with the resolution of 1 cm^−1^. The hydrogen bonding index (R) was calculated with data obtained by deconvolution of FTIR spectra in the carbonyl group range into Gaussian components by Origin 8.5 Pro software (OriginLab Corp., Northampton, MA, USA), according to the following equation:

(1) $$R_{C=O}=\frac{A_{1}+A_{2}}{A_{3}+A_{4}}$$

where: *A*_1_ and *A*_2_ are the total area of the peaks obtained as a result of deconvolution of the FTIR spectrum in the range 1680–1630 cm^−1^ and 1727–1705 cm^−1^ for hydrogen bond of carbonyl groups of urea and urethane, respectively, while *A*_3_ and *A*_4_ are the sum areas of the peaks obtained by deconvolution in the wavelength range 1701–1690 cm^−1^ and 1760–1736 cm^−1^ relating to vibrations of unbounded with the hydrogen bond of the carbonyl groups of urea and urethane groups [4]. Additionally, the degree of phase separation (DPS) parameter was calculated according to the following formula:

(2) $$DPS=\frac{R_{C=O}}{R_{C=O}+1}$$

The content of closed cells in the samples was measured in accordance with ISO 4590. The cell structure was examined with the use of an optical microscope with VideoKit, whereas the Aphelion TM program enabled an analysis of images.

The apparent density was determined in accordance with PN-EN ISO 845 with an accuracy of 0.01 g and 0.1 mm.

The heat conduction coefficient tests were carried out using foam samples with dimensions of 200 × 200 × 50 mm and a FOX 200 apparatus produced in accordance with the ISO 8301 standard at an average temperature of 10 °C. During the measurement, the temperature of the cold plate was 0 °C and the temperature of the warm plate was 20 °C.

The compressive strength was measured at 10% deformation of foams in accordance with the PN-EN ISO 844 standard using a Zwick Roell Z005 (Zwick Roell Group, Ulm, Germany). For each foam, five samples with a diameter and length of 33 mm were analyzed, both perpendicular and parallel to the direction of foam growth. The compressive force was applied at a speed of 2 mm/s.

The dimensional stability was tested after 24 h conditioning of the PU samples at a temperature of +70 °C. Changes in linear dimensions were calculated according to the formulas of the PN-EN 1604+AC standard. In this standard, the designations were taken as $∆\varepsilon_{l}$—change in sample length, $∆\varepsilon_{b}$—change in sample width, $∆\varepsilon_{d}$—change sample thickness in per cent.

The thermal properties of PU materials were examined by thermogravimetric analysis (TGA) with the temperature set between 25 °C and 900 °C at a heating rate of 10 °C·min^−1^ under nitrogen atmospheres using a TG 209 F1 Netzsch (apparatus). 10 mg ± 0.1 mg samples were placed in ceramic pans. The initial decomposition temperature Ti was determined as the temperature at which the mass loss was 5%.

## 3. Results and Discussion

### 3.1. Effect of Water Content

The works described in the literature regarding the content of water as a chemical foaming agent refer to closed-cell PU foams. This part of the work presents the effect of water on the properties of open-cell PU foams with an isocyanate index of 1.1. Water was used in an amount of 20–28 wt.% based on the weight of the modified cooking oil bio-polyol.

**Figure 1:**
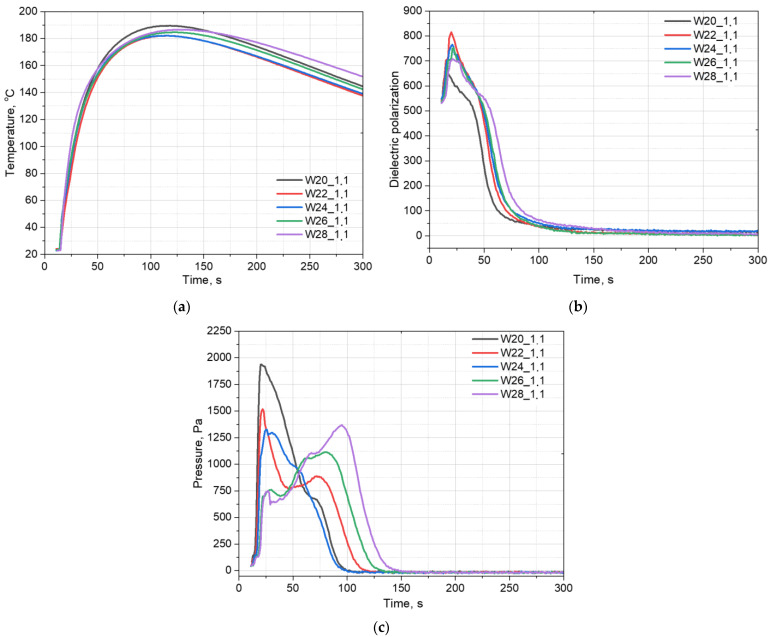
The influence of the water content on the system temperature (**a**), dielectric polarization (**b**), and pressure (**c**) during the foaming process.

A change in the water content in the composition for the production of PU foams can significantly affect the course of the foaming process. Figure 1 shows the temperature (a), dielectric polarization (b), and pressure changes (c) over time, depending on the amount of water in the system. It was observed that the temperature in the foam core was approximately the same in all the analyzed systems and was in the range of 180–190 °C.

Dielectric polarization is a key parameter that gives an insight into electrochemical processes during the transition from a mixture of liquid components, such as polyol, isocyanate, and additives, to the final PU foam. Polyols and isocyanates are characterized by a strong dipole moment, unlike the resulting PUs [13]. The observation of the curves corresponding to the change in dielectric polarization during polyurethane formation shows that increasing the amount of water in the PU system reduced the rate of the reactions taking place (Figure 1b). The more water in the system, the slower the dielectric polarization value decreased.

The greatest differences across the systems can be observed in the course of the pressure exerted on the bottom of the mold by a growing PU foam (Figure 1c). It was observed that the maximum pressure value was reached after a longer time for the foams that contained a higher amount of water. This is due to a slower process of PU bond formation, and, thus, cross-linking. In addition, in the case of the systems containing a greater amount of the water as a chemical blowing agent, faster partial opening of the foam cells was observed. For the systems containing 20% of water, no partial cracking of the cells in the rise foam was observed, the pressure rose to its maximum value, and then dropped back to zero.

**Figure 2:**
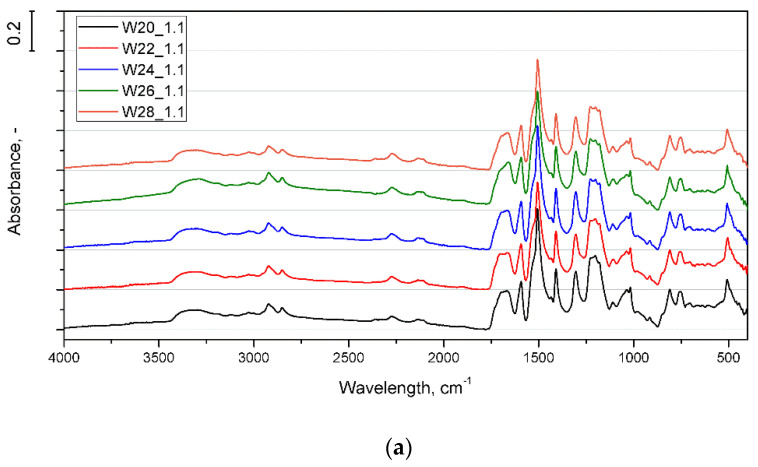

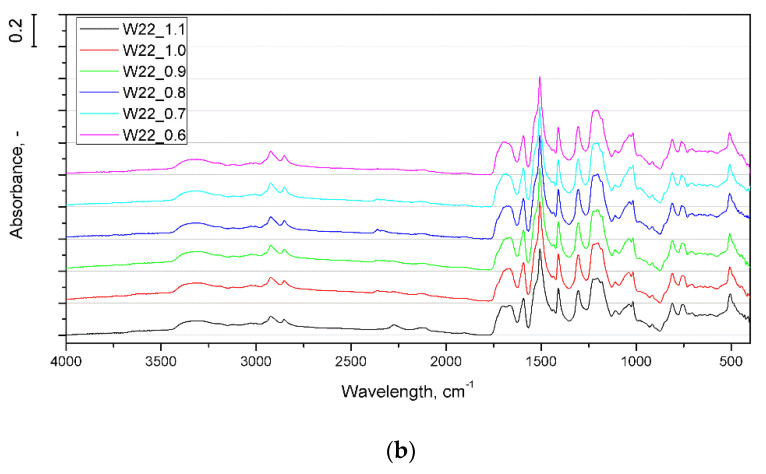
FTIR spectra of PUR foams obtained with different amounts of water (**a**) and different NCO indices (**b**).

Figure 2 shows the FTIR spectra obtained for foams made with different amounts of water (Figure 2a) and different isocyanate indices (Figure 2b). The spectra consist of absorption bands typical for rigid PU, corresponding to N-H stretching (3336 cm^−1^) confirming that urethane groups were successfully formed, C-H stretching (2924 cm^−1^), O-CH_2_ stretching vibrations (2852 cm^−1^), hydrogen-bonded urethane and urea (1715, 1654 cm^−^^1^), C=O stretching vibrations in the amide I region (1704 cm^−1^), Ar-H deformation (1592 cm^−1^), N-H deformations (1510 cm^−1^), CH_3_ deformations (1411 cm^−1^), coupled C-N and C-O stretching vibrations (1174 cm^−1^), C-O-C ester deformation (1220, cm^−1^), and CH deformations of aromatic groups (800–600 cm^−1^) [25,26].

In the case of material series manufactured with different amounts of water and with the isocyanate index of 1.1, absorption peaks at 2275 cm^−1^ and double peaks at 2138 and 2112 cm^−1^ were observed. The first may refer to unreacted isocyanate groups [25,26], while the second is related to forming of carbodiimide functional groups [27]. Those absorption bands were not noted for foams with a lower isocyanate indices.

**Figure 3:**
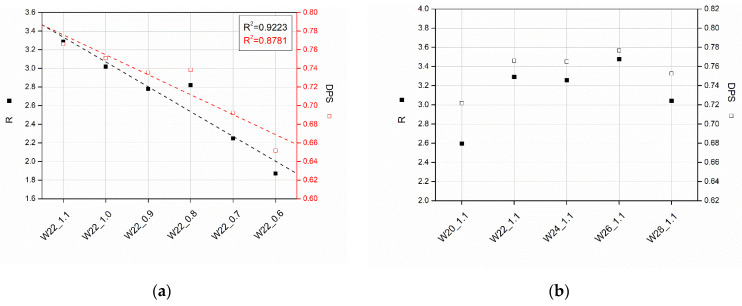
Hydrogen bonding index (R) and degree of phase separation (DPS) calculated on the basis of FTIR spectra for foams obtained with different amounts of water (**a**) and different NCO indices (**b**).

Figure 3 shows the results of the calculated R_C=O_ and DPS, according to Equations (1) and (2) for PU foams made with the addition of various amounts of water and different isocyanate indices. From the data obtained for foams produced with a variable NCO index, it can be concluded that increasing the isocyanate content in the composition increases the hydrogen bonding index and the associated degree of phase separation. Increasing the isocyanate concentration resulted in a more significant amount of hydrogen bonds, the number of rigid segments, and increased cross-link density of the foams. The reduced content of DPS can be interpreted as a lower amount of hydrogen bonds in the material produced with insufficient isocyanate [28]. In the case of a change in the water concentration in the composition, it can be observed that only for the highest and lowest water content observable changes in the DPS have been noted. While the influence of the addition of water on the PU microstructures cannot be under-estimated, this factor has a much smaller impact on its changes compared to the impact of the isocyanate index.

**Figure 4:**
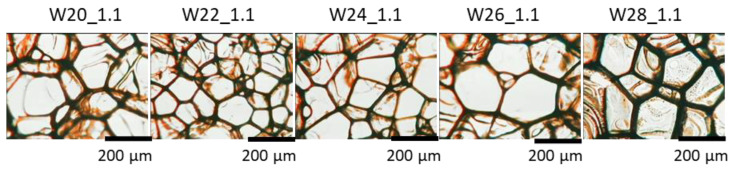
The cellular structure of the foams modified with different amounts of the water as foaming agent examined with the use of an optical microscope.

Among the most important parameters influencing the mechanical and thermal insulation properties of PU foams are the cell structure and apparent density. The influence of the water content in PU systems on the size, shape, and content of cells is shown in Figure 4 and Table 3. The foam with 22 g of water had the smallest cells and the highest cell density, while the foam with 26 g of water produced the cells with the largest sizes. However, the amount of water did not affect the shape of cells, as the anisotropy coefficient is at a similar level. The bio-foams with different contents of the water were characterized by an open-cell structure because the content of closed cells was lower than 5%.

**Figure 5:**
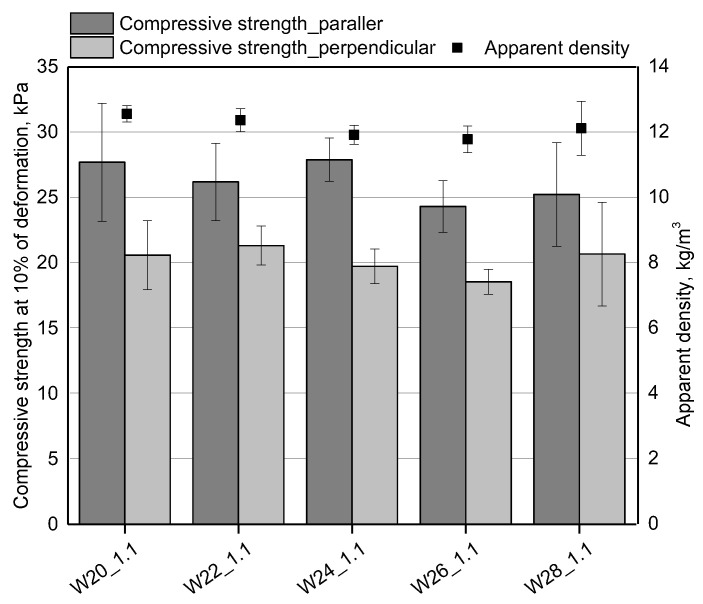
The apparent density and compressive strength of open-cell PUR foams obtained with different amounts of water.

Figure 5 shows that increasing the water amount from 20 to 26 wt.% in relation to 100 g of bio-polyol causes a slight reduction of the foam’s apparent density. This effect is associated with an increase in the content of the carbon dioxide released, which is formed as a result of the reaction of water with isocyanate. The more water used in the synthesis, the more gas is released, thus lowering the apparent density. From the economic point of view, a low apparent density of applied foams is an advantage, however, a higher amount of water is used, and a higher amount of expensive isocyanate has to be used to keep the isocyanate index. In our experiments, an exception was the foam with 28 g of water. In the case of this material, a slight increase in the apparent density was observed, which may be related to a high content of the foaming agent and, consequently, a disturbed foaming process. A confirmation of an inhomogeneous structure the W28_1.1 foam is also a high standard deviation of the apparent density compared to the results for other materials.

In the case of open-cell PU foams, the compressive strength is about 10 kPa [4,13]. It is significantly determined by the cell structure, and apparent density. In our work, the compressive strength measurements taken in the direction parallel to the foam growth direction are higher than the values for the perpendicular direction (Figure 5). The differences may be related to the anisotropic cell structure resulting from the elongation of cells towards the growth of the foam material [17]. There was also a tendency for the compressive strength to decrease with increasing the amount of water from 20 to 26 wt.% in relation to the bio-polyol. The compressive strength in the parallel and perpendicular directions decreased by an average of 3.5 kPa with an increase in the amount of water. The decrease of the compressive strength can be related to the decrease of the apparent density. For the foam with 26 g of water, the compressive strength was the lowest in both measurement directions up to the foam growth. For the material with a 28 wt.% water content in relation to the bio-polyol, a slight increase in the compressive strength was observed and it could be related to the higher apparent density that characterized the foam W28_1.1.

**Figure 6:**
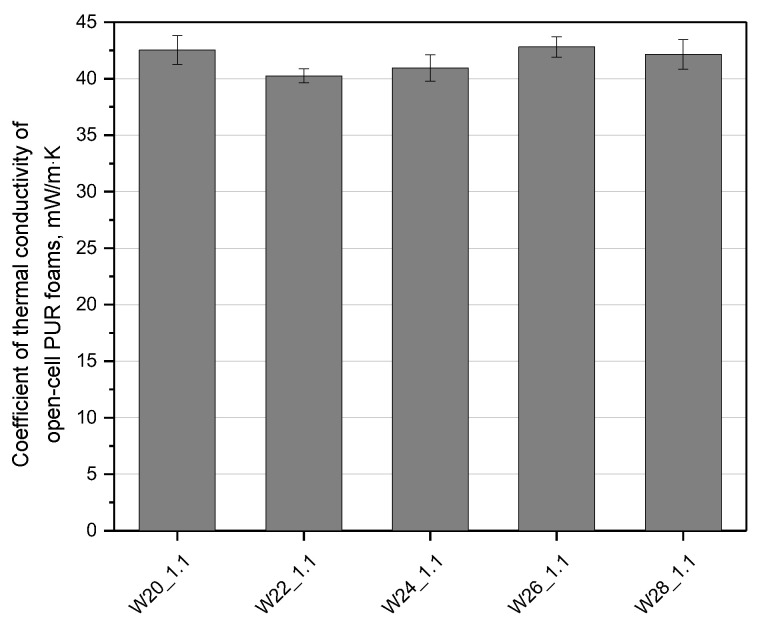
The coefficient of thermal conductivity of the open-cell PU foams with different amounts of water.

Open-cell PU foams, despite slightly higher values of thermal conductivity than their closed-cell counterparts, can still be used as thermal insulation materials. The effects of changing the amount of water on the thermal conductivity in the foams described here are shown in Figure 6. The bio-foams had average thermal conductivity of about 41.3 mW/M·K, which are comparable to those of commercial open-cell PU foams [4]. Carbon dioxide, which is produced in the reaction between isocyanate and water and is characterized by a low value of thermal conductivity coefficient, is mainly responsible for relatively good thermal insulation properties of bio-foams with closed cells [24]. The thermal conductivity of the open-cell foams studied here is affected by their cell structure as the foaming gases are replaced with air. The foam with the addition of 22 wt.% of water in relation to the mass of the bio-polyol was characterized by the lowest value of the thermal conductivity coefficient. This effect can be related to the fine-cell structure of this material. As expected, in the case of the W26_1.1 foam, the value of the thermal conductivity was the highest, which was related to the least favorable cellular structure.

Dimensional stability of PU foams is directly related to their compressive strength [16]. In the experiment discussed here, despite of the deterioration of the mechanical properties along with the increase in the amount of water, the dimensional stability at 70 °C for all obtained materials was satisfactory. The mean percentage changes of linear quantities were lower than 0.6% regardless of the measurement direction (Table 4). Slight changes in the dimensional stability are related to the fact that for open-cell PU foams, the differences between the pressure in the foam cells and the external atmospheric pressure are close to 0 [19].

### 3.2. Effect of Isocyanate Index

For industrial purposes, open-cell and closed-cell PU foams are often sprayed with a spray gun under high pressure. Polyol premix and isocyanate are usually dosed in a 1:1 volume ratio [29]. In order to ensure such conditions, the amount of isocyanate is most often changed, and, as a consequence, the associated isocyanate index changes. The results presented below show the effect of the change in the isocyanate index on the properties of the open-cell PU foams obtained with the use of bio-polyol. The isocyanate index was lowered from 1.1 to 0.6. In all the foams water was introduced in a constant amount of 22 parts/100 parts of bio-polyol.

**Figure 7:**
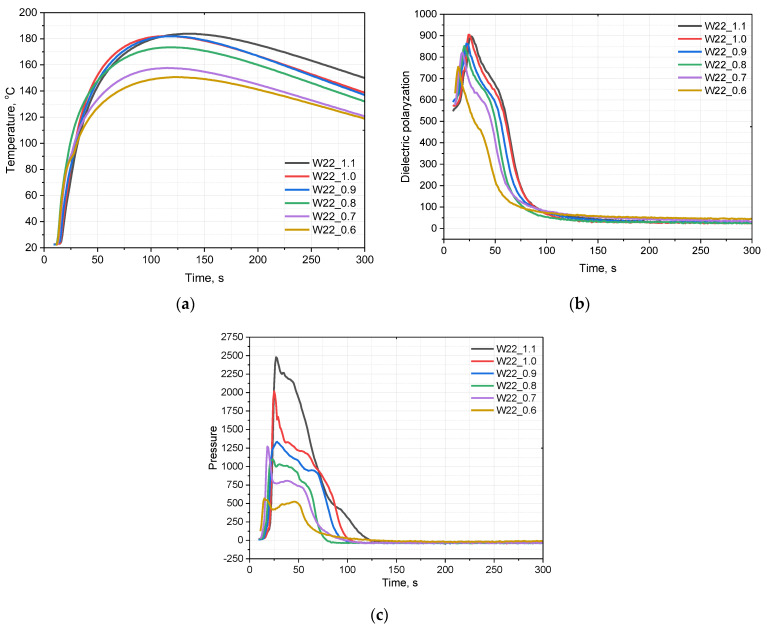
The influence of the isocyanate index on the system temperature (**a**), dielectric polarization (**b**), and pressure (**c**) during the foaming process.

The analysis of the foaming process shows that its course is significantly affected by the isocyanate index. Figure 7a presents the temperature change in the core of the foam obtained from systems with different isocyanate indices. It was observed that with a decreasing isocyanate index, the core temperature of the foam also decreased. This is due to the fact that the reaction of isocyanate with water is highly exothermic [23], therefore, in systems with more isocyanate components, greater amounts of heat are released.

Figure 7b shows the change in the value of dielectric polarization during the foaming process for the materials obtained with different isocyanate indices. The course of the polarization curves is similar for all the systems, however, a slight slowdown in the decrease of the dielectric polarization value can be observed, which is caused by the presence of a larger number of isocyanate groups with a high dipole moment.

The pressure during the foaming process is defined as the pressure of the foamed materials exerted on the substrate. Such an approach reflects in a very good way the impact of the material undergoing the foaming process on the mold walls. The changes of isocyanate index influence the dimensional stability of the resulting material. The higher the isocyanate content, the more rigid polyurethane is obtained, and the gelation time is shortened [30]. The result is that the gas is kept inside cells and the pressure exerted on the walls by the gas does not deform them. In the case of longer gelling times and more flexible polyurethane walls, the pressure exerted by the gas is more easily transferred to the measuring table.

**Figure 8:**
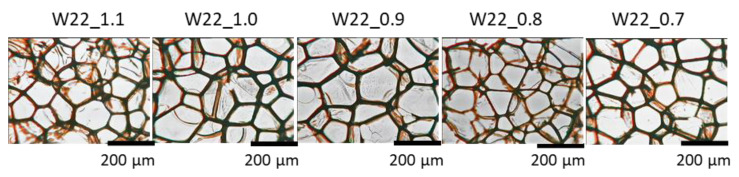
The cellular structure of the foams with different NCO indices examined with the use of an optical microscope.

**Figure 9:**
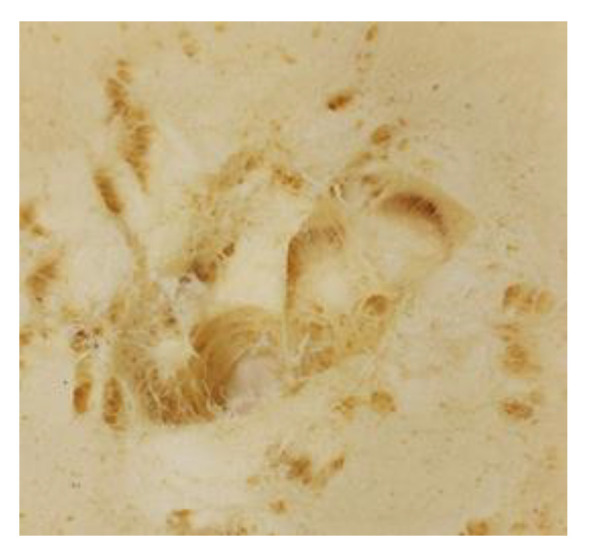
Disturbances in the cellular structure of the foam W22_0.6.

Table 5 and Figure 8 show the important effect of the change in the isocyanate index on the cellular structure of the foams. The foam with the isocyanate index 0.6 was characterized by a disturbed cell structure (Figure 9), which made it impossible to study its cell structure and physico-mechanical properties. In the entire analyzed range of the isocyanate index, the content of closed cells in the foams was lower than 5% (2% on average). Thus, all obtained bio-foams were open-celled. While the foam with the highest isocyanate index (1.1) was characterized by the most isotropic shape among the synthesized materials, the foam with the isocyanate index of 0.8 had the highest cell density, and, thus, the most fine cells. Small foam cell sizes can be an effect of the most dynamic foaming process of confirmed by the highest temperature increase in the beginning of foaming process, the shortest times of cell opening (pressure decreasing). Such cellular structure, among other things, beneficially influenced thermal insulation properties of this foam.

**Figure 10:**
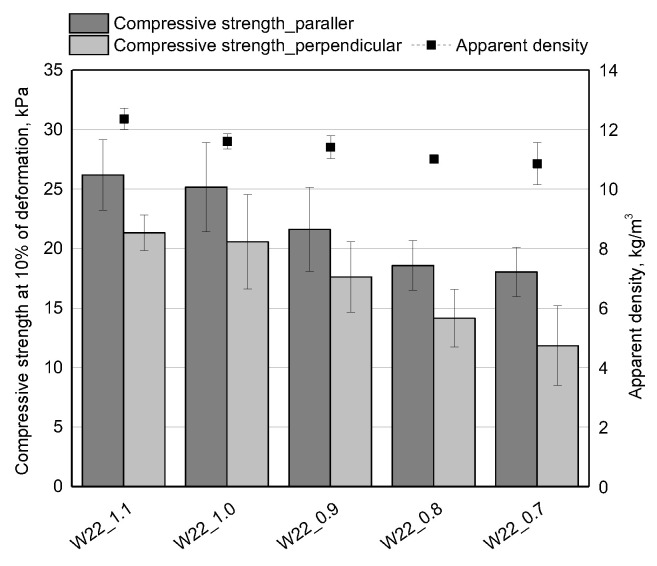
The apparent density and compressive strength of the open-cell PUR foams with different NCO indices.

Along with the decrease of the isocyanate index, the apparent density of the open-cell PU foams was reduced from 12.5 to about 10.0 kg/m^3^ (Figure 10).

Introducing a constant amount of water into the system, despite the decrease in the mass of isocyanate, caused the amount of released CO_2_ not to change, because the isocyanate did not limit the reaction with water. Thus, the constant mass of released gas and the lower mass of the foam system result in the observed effect of decreasing apparent density of open-cell PUR bio-foams. Decreasing the mass of isocyanate in the polymerization process resulted in a decrease in the total density of the polymer network [31]. H. Fan and his team observed a similar impact of the NCO index in their work [31]. However, the research at that time concerned closed-cell foams.

The apparent density and cellular structure also significantly influenced the compressive strength of the foams (Figure 10). It has been found that the anisotropic cellular structure influences the mechanical properties of open-cell PU foams [17]. Due to the anisotropic cell structure, the compressive strength was measured in two directions: parallel and perpendicular to the direction of foam rise. The compressive strength in the direction perpendicular to the foam rise is lower than for the parallel direction for each value of the NCO index. The value of compressive strength in perpendicular direction decreased from 21 kPa to about 13 kPa for foam with NCO index of 0.7. The compressive strength measured in the direction parallel to the foam growth decreased from an average of about 27 kPa for an NCO index of 1.1 to about 18 kPa for an NCO index of 0.7. The effect of decreasing compressive strength is related to a lower content of isocyanate groups that were introduced into the foam system in response to a lower NCO index. Decreasing the content of isocyanate groups resulted in a decrease in hard segments content in the structure of PU foams. The lower compressive strength of the foams with lower isocyanate indices was also a result of the lower apparent density of these materials.

Table 6 summarizes the results of the measurements of the dimensional stability of the open-cell PU foams depending on the NCO index. It was noted that despite the change in the NCO index from 1.1 to 0.7, the mean values of dimensional stability were at a similar level—lower than 0.15%—regardless of the measurement direction. This means that all the PU bio-foams were characterized by good dimensional stability.

**Figure 11:**
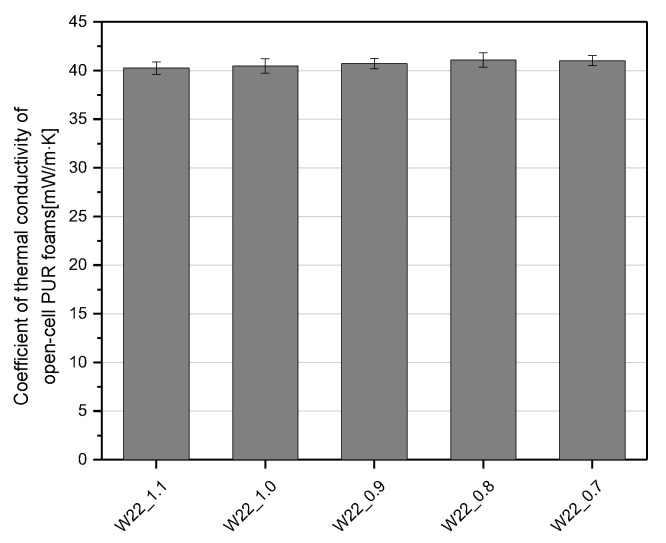
The coefficient of thermal conductivity of the open-cell PUR foams with different NCO indices.

Figure 11 shows how the change in the NCO index from 1.1 to 0.7 influenced the values of thermal conductivity. The open-cell bio-foams were characterized by an average thermal conductivity coefficient in the range of 40.0–43 mW/M·K.

Regardless of the isocyanate index used, the values of the thermal conductivity coefficient were characterized by similar values. The authors found no significant influence of the isocyanate index on thermal insulation properties.

### 3.3. Thermal Stability of PU Foams

**Figure 12:**
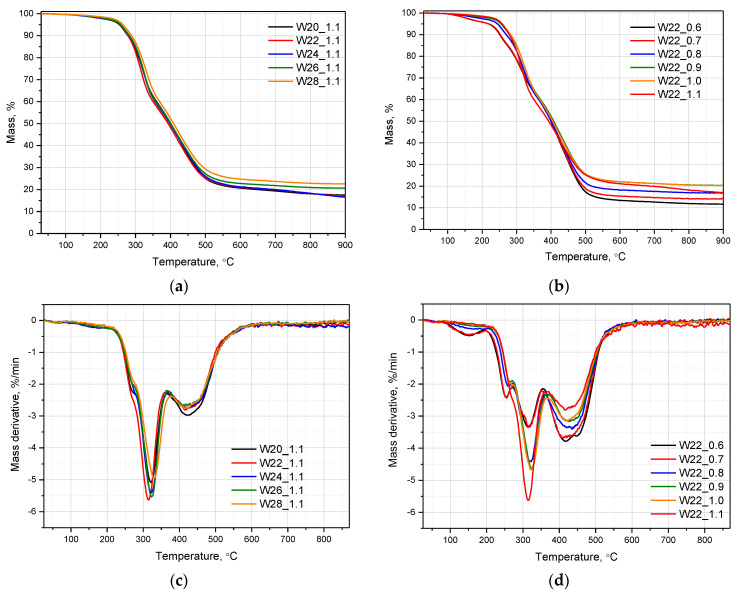
TG and DTG curves of polyurethane open-cell PU foams with different amounts of chemical blowing agent (**a**,**c**), and different isocyanate index (**b**,**d**).

Figure 12 shows the results of the thermogravimetric analysis; TG and DTG curves are presented separately for the PU series with different water contents and the series made with a constant water content and a variable isocyanate index. Additional data allowing a quantitative comparison of the changes in the thermal stability of the materials related to temperature with a 5, 10, 20, and 50% mass loss (T5%, T10%, T20%, and T50%) and residuals at the maximum temperature are collectively presented in Table 7. In the case of the DTG curves enabling a detailed analysis of individual steps of degradation, it can be observed that in the case of an increasing share of isocyanate in the composition, the curve course is simplified. The PU becomes more thermally stable with increasing isocyanate index. Three-step degradation for W22_1.1 changed into five-step degradation process observed for W22_06. The first degradation step, followed only for series with the lowest isocyanate index (W22_0.6, and W22_0.8), in the range of 90–190 °C, corresponded to decomposition of the unreacted with isocyanate low molecular weight compounds from TEA polyol, including unreacted acid [32], or may have reflected unreacted water evaporation [33,34]. The second peak, dominant in the DTG curves, visible for all samples in the range of 195–360 °C, consisted of two overlapping peaks with maxima at about 250–260 °C and about 318 °C related to the degradation of urethane bonds in the rigid segment of polyurethane [35]. The maxima observed in the range of 370–420 °C corresponded to the degradation of ester bonds in polyols and urea bonds [32,36]. Most of the samples revealed one peak at 421 °C in the range of 367–550 °C; for a series with the lowest isocyanate index, two peaks at 412 °C and 452 °C were seen. The peaks resulted from the degradation of aromatic compounds and char products. A similar reduction in the peak value in this range as an effect of the increased isocyanate index was reported by Kirpluks et al. [37].

The analysis of the TG curves showed that the change in the isocyanate index had a significantly greater influence on the differentiation of the thermal stability of polyurethanes than the variable water content at a constant NCO index of 1.1. The increasing isocyanate content in the composition resulted in a gradual improvement in thermal stability. Moreover, the difference at the beginning of the thermal decomposition assessed by T5% between the lowest and highest isocyanate index characterized PUs was more than 39 °C. This thermal parameter is mainly influenced by the disappearance of the first-step decomposition mentioned earlier observed in the range of 80–190 °C. Moreover, the increasing NCO index resulted in a significantly higher residual mass at 900 °C. The TGA analyzes showed almost no changes in the course of the TG curve of the PU foams with different water contents. However, in the case of the foams containing 26 and 28 g of water, an increase in thermal stability was observed at higher temperatures, which may be a result of a higher amount of urea bonds in the cross-linked structure. These materials were also characterized by a higher residue at a temperature of 900 °C. In the case of all samples, there was a shoulder inflection of the DTG curve at 280 °C, and the shifts of the main urethane degradation step peak showed no relationship to the water content, assuming that the content of the polyol was constant in the series of the PU foams made with various contents of the water. Therefore, the increase of temperature at 50% of mass loss may be indirectly related to the increasing amount of urea bonds (Table 7).

## 4. Conclusions

This paper describes the influence of water as a chemical blowing agent and the isocyanate index on the cell structure and selected properties of open-cell polyurethane foams. To improve climate neutrality, a polyol derived from modified used rapeseed cooking oil was used for the synthesis of bio-foams.

Increasing the water content in the synthesis of open-cell porous materials resulted in a decrease in the apparent density due to the increased amount of the carbon dioxide released. The gas (with a low value of thermal conductivity coefficient) and the structure with small cell sizes contribute to a relatively low thermal conductivity of the polyurethane foams of around 40 mW/m·K. In consequence of the anisotropic structure of the cells, the compressive strength values in the direction parallel to the foam growth direction were higher than perpendicularly. On the other hand, with the increase in the amount of the chemical foaming agent, a tendency of the compressive strength to decrease was observed, which resulted from a decrease in the apparent density of the foams. The amount of water was not found to influence the dimensional stability of the open-cell polyurethane foams.

In the next stage of the research, the open-cell foams with a lower apparent density were obtained by decreasing the isocyanate index. Slight changes in the thermal conductivity related to the cell structure of the foams were observed.

The decrease in the compressive strength of the open-cell polyurethane foams due to the decrease in the isocyanate index was directly related to the apparent density of the tested materials. All the foams were characterized by very good dimensional stability. No significant effect of isocyanate index changes on the linear dimensions of the foams exposed to a temperature of 70 °C was observed. 

## Figures and Tables

**Table 1:** The formulations of the foams with various water contents.

Component, g	W20_1.1	W22_1.1	W24_1.1	W26_1.1	W28_1.1
Bio-polyol TEA	100.0	100.0	100.0	100.0	100.0
Polycat 142	5.0	5.0	5.0	5.0	5.0
Tegostab 8870	2.0	2.0	2.0	2.0	2.0
Tegostab 8523	0.6	0.6	0.6	0.6	0.6
Ortegol 500	1.0	1.0	1.0	1.0	1.0
TEP	20.0	20.0	20.0	20.0	20.0
Dabco EM400	2.0	2.0	2.0	2.0	2.0
Water	20.0	22.0	24.0	26.0	28.0
PMDI	431.8	464.9	498.1	531.2	564.3
Isocyanate index	1.1	1.1	1.1	1.1	1.1

**Table 2:** The formulations of the foams with different isocyanate indices.

Component, g	W22_1.1	W22_1.0	W22_0.9	W22_0.8	W22_0.7	W22_0.6
Bio-polyol TEA	100.0	100.0	100.0	100.0	100.0	100.0
Polycat 142	5.0	5.0	5.0	5.0	5.0	5.0
Tegostab 8870	2.0	2.0	2.0	2.0	2.0	2.0
Tegostab 8523	0.6	0.6	0.6	0.6	0.6	0.6
Ortegol 500	1.0	1.0	1.0	1.0	1.0	1.0
TEP	20.0	20.0	20.0	20.0	20.0	20.0
Dabco EM400	2.0	2.0	2.0	2.0	2.0	2.0
Water	22.0	22.0	22.0	22.0	22.0	22.0
PMDI	464.9	422.7	380.4	338.1	295.2	253.6
Isocyanate index	1.1	1.0	0.9	0.8	0.7	0.6

**Table 3:** Characteristics of the cell structure of PU foams.

System Symbol	Number of Cells per cm^2^	Cross-Section Average Cell Area [mm^2^]	Anisotropy Index	Cell Density, Number of Cells per cm^3^ × 10^5^	Content of Closed Cells, %
W20_1.1	1740 ± 255	0.0319 ± 0.0040	0.94 ± 0.05	0.73 ± 0.17	<5
W22_1.1	2430 ± 370	0.0253 ± 0.0047	0.95 ± 0.03	1.21 ± 0.28	<5
W24_1.1	1761 ± 438	0.0328 ± 0.0071	0.95 ± 0.11	0.75 ± 0.28	<5
W26_1.1	1625 ± 206	0.0334 ± 0.0033	0.94 ± 0.08	0.66 ± 0.12	<5
W28_1.1	2121 ± 194	0.0263 ± 0.0024	0.90 ± 0.11	0.98 ± 0.13	<5

**Table 4:** The dimensional stability of the bio-foams measured at 70 °C after 24 h.

System Symbol	∆*ε_l_* [%]	∆*ε_b_* [%]	∆*ε_d_* [%]
W20_1.1	−0.08 ± 0.12	−0.09 ± 0.24	−0.50 ± 0.99
W22_1.1	−0.14 ± 0.12	−0.17 ± 0.12	−0.49 ± 0.37
W24_1.1	−0.13 ± 0.15	−0.24 ± 0.17	−0.58 ± 0.44
W26_1.1	−0.15 ± 0.11	−0.16 ± 0.13	−0.46 ± 0.47
W28_1.1	−0.08 ± 0.14	−0.17 ± 0.18	−0.50 ± 0.59

**Table 5:** Characteristics of the cellular structure of PU foams with different isocyanate indices.

System Symbol	Number of Cells per cm^2^	Cross-Section Average Cell Area [mm^2^]	Anisotropy Index	Cell Density, Number of Cells per cm^3^ × 10^5^	Content of Closed Cells, %
W22_1.1	2430 ± 370	0.0181 ± 0.0047	0.95 ± 0.03	1.21 ± 0.28	<5
W22_1.0	2191 ± 148	0.0268 ± 0.0028	0.90 ± 0.04	1.03 ± 0.10	<5
W22_0.9	2260 ± 326	0.0252 ± 0.0044	0.86 ± 0.07	1.08 ± 0.23	<5
W22_0.8	2758 ± 343	0.0214 ± 0.0044	0.88 ± 0.04	1.46 ± 0.28	<5
W22_0.7	2306 ± 282	0.0245 ± 0.0031	0.90 ± 0.08	1.11 ± 0.21	<5

**Table 6:** The dimensional stability of the bio-foams, measured at 70 °C after 24 h.

System Symbol	∆*ε_l_* [%]	∆*ε_b_* [%]	∆*ε_d_* [%]
W22_1.1	−0.14 ± 0.12	−0.17 ± 0.12	−0.49 ± 0.37
W22_1.0	0.00 ± 0.07	0.03 ± 0.10	0.08 ± 0.32
W22_0.9	0.04 ± 0.10	0.02 ± 0.11	0.43 ± 0.37
W22_0.8	0.07 ± 0.12	0.10 ± 0.09	0.08 ± 0.24
W22_0.7	−0.04 ± 0.12	−0.03 ± 0.10	0.40 ± 0.51

**Table 7:** Thermal properties of PU foams obtained by TGA.

System Symbol	T_5%_ [°C]	T_10%_ [°C]	T_20%_ [°C]	T_50%_ [°C]	Residual Mass [%]
W20_1.1	254.2	278.1	309.6	400.7	17.5
W22_1.1	257.6	278.7	305.0	393.1	16.63
W24_1.1	256.8	281.8	310.6	398.4	16.69
W26_1.1	257.1	282.7	312.0	400.5	20.54
W28_1.1	263.8	289.0	318.0	408.7	22.56
W22_1.0	257.7	282.6	312.7	404.7	20.28
W22_0.9	254.4	280.7	312.6	407.4	20.33
W22_0.8	244.1	269.9	306.5	400.0	17.03
W22_0.7	218.4	252.4	295.3	400.4	14.03
W22_0.6	218.1	251.1	294.2	399.7	11.63

## Data Availability

The data presented in this study are available on request from the corresponding author.
